# Effects of blood flow restrictiontraining on muscle function and balance in chronic ankle instability: a systematic review

**DOI:** 10.3389/fphys.2025.1683438

**Published:** 2025-11-18

**Authors:** Ziliang Wen, Bing Zheng, Jiang Zhu, Xuelian Wu, Zonghui Wu

**Affiliations:** 1 Sport Rehabilitation Research Institute of Southwest University, Southwest University, Chongqing, China; 2 Southwest University Hospital, Southwest University, Chongqing, China

**Keywords:** chronic ankle instability, blood flow restriction training, muscle function, balance, systematic review

## Abstract

**Background:**

Chronic ankle instability (CAI) impairs peri-ankle strength and balance. While blood flow restriction training (BFRT) enhances muscle strength, hypertrophy, and activation, its efficacy in CAI remains uncertain, warranting this systematic review.

**Methods:**

This systematic review analyzed randomized controlled trials with BFRT interventions from five databases (PubMed, Scopus, Web of Science, EBSCOhost, CNKI). Relevant data were extracted, and the PEDro Scale was used to assess the methodological quality of each study.

**Results:**

Nine studies from four countries were included, involving a total of 263 patients, with publication dates ranging from 2020 to 2024. The PEDro scores of these studies ranged from 6 to 10. Two of the studies demonstrated positive effects on muscle hypertrophy, five showed significant improvements in muscle strength, and four reported enhanced muscle group activation, although there were variations in the activation of specific muscle groups. Of the seven studies assessing balance, one failed to confirm a positive effect.

**Conclusion:**

This systematic review demonstrates that blood flow restriction therapy (BFRT) combined with low-intensity conventional rehabilitation training significantly enhances ankle muscle strength and promotes muscle hypertrophy in patients with chronic ankle instability (CAI). Despite variations in training protocols and BFRT parameters across studies, these benefits have been consistently observed in both acute (single-treatment) and short-term (4–6 weeks) interventions. Additionally, most of the included studies underscore the beneficial effects of BFRT on improving muscle activation and balance. However, some of the research results are still inconsistent and require further study.

**Systematic Review Registration:**

http://inplasy.com, identifier: INPLASY202490117.

## 1 Introduction

Ankle sprains are among the most prevalent sports-related injuries, commonly observed in competitive sports, military training, and everyday activities (Doherty et al., 2014). Surveys reveal that up to 70% of the general population have experienced an ankle injury during their lifetime. These injuries, second only to knee injuries in terms of prevalence (Liu N. et al., 2024), frequently occur during direct impacts or landing movements, with incidence rates ranging between 20% and 50%, particularly in sports such as basketball, soccer, and volleyball (Kobayashi and Gamada, 2014; Yin et al., 2022). In countries like the United States and the Netherlands, ankle injuries contribute significantly to healthcare expenditures, placing a considerable burden on medical systems (Lin et al., 2021). Additionally, ankle sprains exhibit the highest recurrence rate among lower limb injuries (Delahunt and Remus, 2019). Approximately 70% of patients do not receive timely treatment following an acute sprain, leading to repeated injuries and, ultimately, the development of chronic ankle instability (CAI) (Gribble et al., 2016).

CAI refers to joint instability following an ankle ligament injury, which commonly leads to unilateral or bilateral recurrent sprains (Hertel and Corbett, 2019), including mechanical ankle instability (MAI) and functional ankle instability (FAI). Repeated sprains caused by joint instability can damage the muscle spindle receptors around the ankle, leading to muscle atrophy, as well as deficits in proprioception and neuromuscular control (Anguish and Sandrey, 2018). Clinically, these deficits present as symptoms such as pain, muscle weakness, reduced balance, impaired neuromuscular coordination, abnormal proprioception, and activity limitations (Vallandingham et al., 2019). Among these, impaired muscle function—including muscle activation, hypertrophy, and strength (Narici and Maffulli, 2010) —along with reduced balance, are key risk factors for ankle re-injury (Hertel and Corbett, 2019).

Currently, rehabilitation strategies for chronic ankle instability (CAI) encompass physical therapy, exercise therapy, taping, and the use of ankle braces (Wen et al., 2023). Among these approaches, exercise therapy has emerged as the primary treatment method due to its non-invasive nature, safety, and effectiveness. Blood flow restriction training (BFRT), a novel intervention within exercise therapy, is frequently combined with low-load exercises. This technique applies pressure to the limb using a compression cuff, achieving effects comparable to high-intensity training in terms of enhancing muscle strength and promoting hypertrophy (Korkmaz et al., 2022; Liu H. et al., 2024). The underlying mechanism may facilitate these adaptive changes by inducing local hypoxia, creating metabolic stress, and activating high-threshold motor units, such as type II muscle fibers (Hwang and Willoughby, 2018). Furthermore, research indicates that BFRT enhances muscle activation and reduces the delay in muscle responses to stimuli, both of which are critical for improving balance in CAI patients (Hall et al., 2018; Liu H. et al., 2024). Given its proven efficacy and safety, BFRT holds considerable promise for the rehabilitation of CAI patients. Consequently, a growing body of research has investigated the effects of BFRT on muscle function and balance in individuals with CAI, highlighting the need for a systematic review to synthesize the current evidence.

## 2 Methodology

### 2.1 Registration and protocol

The protocol for this systematic review adhered to the guidelines set forth by the Preferred Reporting Items for Systematic Reviews and Meta-Analyses (PRISMA) (Cao et al., 2024) and was registered in INPLASY with the registration number INPLASY202490117.

### 2.2 Eligibility criteria

The following inclusion criteria were set according to the PICOS framework:Population, 1) Participants were patients with chronic ankle instability, with no restrictions on gender or age; Intervention, 2) The experimental group performed conventional rehabilitation training with the addition of BFRT via a pressurized cuff; Comparison, 3) Sham blood flow restriction training (where cuffs were worn but no pressure was applied) or conventional rehabilitation served as the control group; Outcome, 4) At least one outcome metric in the study that included muscle strength, muscle activation, muscle hypertrophy, and balance; Study type, 5) Randomized controlled trials of two or more groups or single group experiments; 6) Full-text studies published in English or Chinese. The exclusion criteria were:1)Surgical treatment and non-BFRT studies; 2) Reviews, conference abstracts, letters to the editor, case reports and newsletters; 3) Studies that are unpublished or for which no valid information can be extracted.

### 2.3 Searching strategy and selection process

The search was conducted on 28 August 2024. The following databases were used: Web of Science, PubMed, China National Knowledge Infrastructure (CNKI), EBSCOhost, Scopus. A Boolean search syntax using the operators“AND”and“OR”was applied. The search terms were“blood flow restriction” OR “blood flow restriction training” OR “blood flow restriction exercise” OR “blood flow restriction therapy” OR “KAATSU Training” OR “vascular occlusion training” AND “chronic ankle instability” OR “functional ankle instability” OR “mechanical ankle instability” OR “ankle instability” OR “ankle” AND “balance” OR “postural stability” OR “postural control” OR “strength training” OR “muscle strength” OR “muscle activation” OR “muscle cross-sectional area” OR “muscle hypertrophy” OR “muscle”.

### 2.4 Study selection

Endnote software (X9, Thomson Reuters, New York City, NY, United States) was used to remove duplicates. Subsequently, two authors (XW and JZ) independently screened the results based on the title and abstract. Then, two authors (ZiW and BZ) reviewed these studies according to the inclusion criteria and PICOS. All processes were determined through discussion, and any discrepancies (e.g., types of intervention, study design) were resolved with consulting the Correspondence author (ZoW) if necessary.

### 2.5 Data extraction

Data extraction from the included studies was independently performed by two authors (ZiW and JZ), which included: (1) participant characteristics (sex, age); BFRT and other interventions; (3) comparison (control group); (4) intervention characteristics(cuff parameters, pressurization pressure, load intensity, training content, program length, frequency, session duration); (5) Assessments (test to measure the effect of BFRT on CAI patients); and (6) outcomes (results from pre-to post-intervention and between-group comparisons). Any disagreement in data extraction was resolved by the fourth author (XW).

### 2.6 Quality assessment

The methodological quality of the included literature was evaluated using the PEDro scale. The scale consists of 11 items, involving the evaluation of four methodological areas: randomization, blinding, group comparison, and data analysis (Moseley et al., 2019). The PEDro scale measures methodological quality on a scale from 0 to 10, with higher scores indicating higher quality: 9–10 denotes outstanding quality; 6–8 denotes good quality; 4–5 denotes medium quality; and below 4 denotes poor quality (Cashin and McAuley, 2020). Two independent researchers (ZW and BZ) performed the quality assessment, and a third researcher was consulted if necessary for disagreement.

## 3 Results

### 3.1 Study selection

**FIGURE 1:**
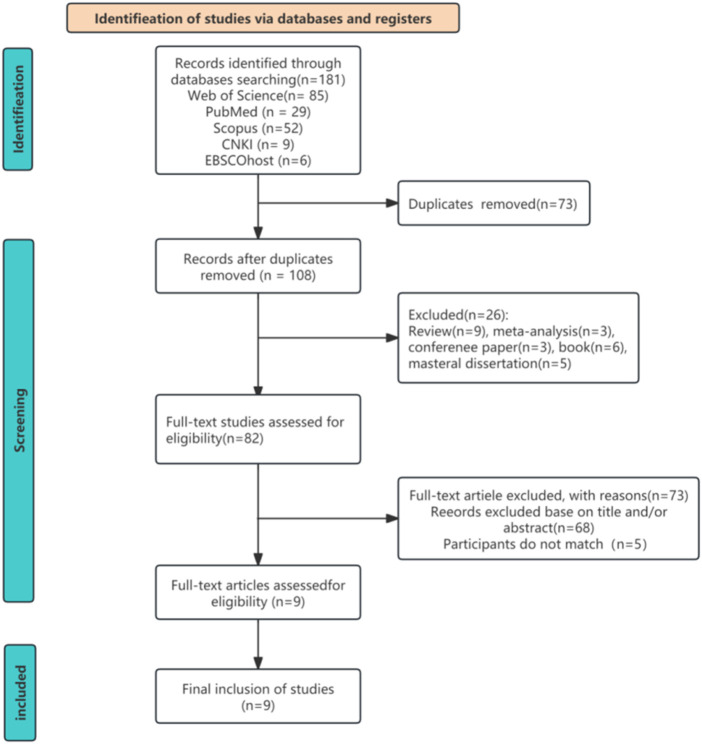
Study selection flow diagram.

A total of 181 studies were initially identified through searches across multiple electronic databases. After removing duplicates, the titles and abstracts of 82 studies were screened to determine their relevance based on the inclusion criteria. Of these, 9 articles fulfilled the eligibility criteria, and their full texts were retrieved for further evaluation. Ultimately, all 9 studies (Killinger et al., 2020; Burkhardt et al., 2021; Werasirirat and Yimlamai, 2022; Mahmoud et al., 2023; Wen et al., 2023; Clark et al., 2024; Liang et al., 2024; Liu S. et al., 2024; Liu and Wang, 2024) were included in this systematic review following thorough assessment. The selection procedure details are illustrated in Figure 1.

### 3.2 Study quality assessment

The PEDro scores varied from 6 to 10 for the studies included in this review (Table 1). With an average score of 7.1, indicating a generally good overall quality of the literature.

**TABLE 1:** PEDro Scale scores of the reviewed articles.

References	0	1	2	3	4	5	6	7	8	9	10	PEDro total score
Killinger et al. (2020)	1	1	1	0	0	0	0	1	1	1	1	6
Burkhardt et al. (2021)	1	1	0	1	0	0	0	1	1	1	1	6
Werasirirat and Yimlamai (2022)	1	1	0	1	1	0	0	1	1	1	1	7
Wen et al. (2023)	1	1	1	1	1	1	1	1	1	1	1	10
Mahmoud et al. (2023)	1	1	1	1	1	1	1	0	1	1	1	9
Liu S. et al. (2024)	1	1	0	1	0	0	1	1	1	1	1	7
Clark et al. (2024)	1	1	0	1	0	0	0	1	1	1	1	6
Liu and Wang (2024)	1	1	0	1	1	0	0	1	1	1	1	7
Liang et al. (2024)	1	1	1	1	0	0	0	0	1	1	1	6

Citeira: 0) Eligibility criteria; 1) Random allocation; 2) Concealed allocation; 3) Groups similar at baseline; 4) Participant blinding; 5) Therapist blinding; 6) Assessor blinding; 7) <15% dropouts; 8) Intention to treat analysis; 9) Between group Difference reported; 10) Point estimate and variability reported.

### 3.3 Participant characteristics

This literature review includes nine randomized controlled trials (Killinger et al., 2020; Burkhardt et al., 2021; Werasirirat and Yimlamai, 2022; Mahmoud et al., 2023; Wen et al., 2023; Clark et al., 2024; Liang et al., 2024; Liu S. et al., 2024; Liu and Wang, 2024). The studies were published between 2020 and 2024, with four conducted in China (Wen et al., 2023; Liang et al., 2024; Liu S. et al., 2024; Liu and Wang, 2024), three in the United States (Killinger et al., 2020; Burkhardt et al., 2021; Clark et al., 2024), one in Thailand (Werasirirat and Yimlamai, 2022), and one in Saudi Arabia (Mahmoud et al., 2023). A total of 263 participants were involved, with sample sizes ranging from 19 to 46, averaging 30 participants per study. All participants were aged between 18 and 30 years. Of the included studies, eight investigated both male and female participants (Killinger et al., 2020; Burkhardt et al., 2021; Werasirirat and Yimlamai, 2022; Wen et al., 2023; Clark et al., 2024; Liang et al., 2024; Liu S. et al., 2024; Liu and Wang, 2024), with only one study focusing on females only (Mahmoud et al., 2023). These characteristics can be seen in Table 2.

**TABLE 2:** General characteristics of the included studies and sample.

References	Year	Country	Study design	Sample	Age (years ± SD)	Sex
Killinger et al. (2020)	2020	United States	RCT	19	20.8 ± 2.3	10F/9M
Burkhardt et al. (2021)	2021	United States	RCT	25	21.8 ± 2.8	10F/15M
Werasirirat and Yimlamai (2022)	2022	Thailand	RCT	16	22 ± 1.03	NR
Wen et al.(2023)	2023	China	RCT	46	EG: 20.50 ± 1.06 CG: 20.50 ± 1.07	NR
Mahmoud et al. (2023)	2023	Saudi Arabia	RCT	39	EG: 22.66 ± 1.82 CG1: 23.33 ± 1.98 CG2: 24.5 ± 2.23	39F
Liu S. et al. (2024)	2024	China	RCT	23	EG: 20.67 ± 1.30 CG: 20.82 ± 1.47	17F/6M
Clark et al. (2024)	2024	United States	RCT	25	20.8 ± 2.3	16F/9M
Liu and Wang (2024)	2024	China	RCT	30	EG: 20.27 ± 1.79 CG: 19.60 ± 1.68	NR
Liang et al. (2024)	2024	China	RCT	40	EG: 21.27 ± 1.90 CG1: 20.18 ± 1.55 CG2: 21.82 ± 2.04	NR

RCT, randomized controlled trial; SD, standard deviation; EG, experimental group; CG, control group; M, male; F, female; NR, not reported.

### 3.4 Intervention characteristics

The characteristics of the intervention programs included in the study are as follows:

#### 3.4.1 BFRT parameter settings

In the nine studies included, eight (Killinger et al., 2020; Burkhardt et al., 2021; Werasirirat and Yimlamai, 2022; Mahmoud et al., 2023; Wen et al., 2023; Liang et al., 2024; Liu S. et al., 2024; Liu and Wang, 2024) utilized cuffs with widths ranging from 5 to 14 cm, while one study did not specify the cuff width. The studies differed in their cuff placement on the lower extremity: six studies (Killinger et al., 2020; Werasirirat and Yimlamai, 2022; Mahmoud et al., 2023; Clark et al., 2024; Liang et al., 2024; Liu S. et al., 2024) placed the cuff on the proximal thigh (inguinal crease region), two studies (Burkhardt et al., 2021; Wen et al., 2023) placed it on the proximal knee, and one study (Liu and Wang, 2024) did not report the cuff placement. Regarding pressurization, six studies (Burkhardt et al., 2021; Werasirirat and Yimlamai, 2022; Mahmoud et al., 2023; Wen et al., 2023; Clark et al., 2024; Liang et al., 2024) used an arterial occlusion pressure (AOP) of 40%–80%, while the remaining three employed an 80% limb occlusion pressure (Killinger et al., 2020), a fixed pressure of 20–50 mmHg (Liu and Wang, 2024), and a 7-point subjective pressure rating (Liu S. et al., 2024), respectively, to restrict blood flow.

#### 3.4.2 Intervention prescriptions

1. Type of Intervention: Among the nine included studies, rehabilitation interventions for CAI patients were classified into four categories. Type 1 focused on resistance training (Killinger et al., 2020; Wen et al., 2023); Type 2 emphasized balance training (Burkhardt et al., 2021; Clark et al., 2024); Type 3 combined both resistance and balance training (Werasirirat and Yimlamai, 2022; Mahmoud et al., 2023; Liang et al., 2024); and Type 4 incorporated resistance and balance training along with physical therapy (Liu S. et al., 2024; Liu and Wang, 2024).
2. Amount of Load: Each exercise in the intervention was typically performed in four sets: the first set consisting of 30 repetitions, followed by three sets of 15 repetitions. Alternatively, some studies employed 3 to 6 sets with the same repetition scheme for each movement.
3. Load Intensity: Four studies (Burkhardt et al., 2021; Werasirirat and Yimlamai, 2022; Mahmoud et al., 2023; Wen et al., 2023) defined load intensity using a percentage of one-repetition maximum (1RM), ranging from 20% to 40%. Another four studies (Clark et al., 2024; Liang et al., 2024; Liu S. et al., 2024; Liu and Wang, 2024) described the training as low-intensity. One study (Killinger et al., 2020) monitored exercise intensity using 30% of the maximum voluntary isometric contraction (MVIC).
4. Intervention Frequency and Duration: Except for three studies (Killinger et al., 2020; Burkhardt et al., 2021; Clark et al., 2024) that investigated the effects of a single exercise session on muscle activation, the remaining six studies (Werasirirat and Yimlamai, 2022; Mahmoud et al., 2023; Wen et al., 2023; Liang et al., 2024; Liu S. et al., 2024; Liu and Wang, 2024) had an intervention frequency of 2–3 times per week, with a duration ranging from 4 to 6 weeks. Notably, only one study included a follow-up at the one-year mark (Liu S. et al., 2024).

### 3.5 Intervention outcomes

#### 3.5.1 Effects of BFRT on muscle activation

Four studies (Killinger et al., 2020; Burkhardt et al., 2021; Liang et al., 2024; Liu S. et al., 2024) used surface electromyography to evaluate the effects of BFRT on ankle muscle activation in patients with CAI. The results indicated significant increases in the activation of the tibialis anterior (TA) (Killinger et al., 2020; Liang et al., 2024; Liu S. et al., 2024), gastrocnemius (Liang et al., 2024), and soleus (Burkhardt et al., 2021) following BFRT. One of the studies showed that the activation levels of the TA and gastrocnemius after BFRT were comparable to those achieved through conventional rehabilitation (Liang et al., 2024), while three other studies found that BFRT led to greater activation of the TA (Killinger et al., 2020; Liu S. et al., 2024) and soleus (Burkhardt et al., 2021) compared to conventional training. However, Burkhardt et al. (2021) reported that BFRT did not significantly activate the TA. Furthermore, all studies indicated that BFRT had limited effects on peroneus longus (PL) activation(Killinger et al., 2020; Burkhardt et al., 2021; Liang et al., 2024; Liu S. et al., 2024).

#### 3.5.2 Effects of BFRT on muscle hypertrophy

Two studies used B-mode ultrasound to investigate the effect of BFRT on ankle muscle hypertrophy in patients with CAI. Werasirirat and Yimlamai (2022) found that after 4 weeks of ankle muscle strength and balance training, the BFRT group showed a significant increase in the cross-sectional area of the gastrocnemius, outperforming the conventional training group. Another study similarly observed that after 6 weeks of BFRT, the thickness of the TA, PL, and triceps surae (TS) in CAI patients significantly increased, yielding rehabilitation results comparable to those of the high-intensity training group (Wen et al., 2023).

#### 3.5.3 Effects of BFRT on muscle strength

Five studies employed handheld dynamometers (Wen et al., 2023; Liu and Wang, 2024), isokinetic dynamometers (Werasirirat and Yimlamai, 2022; Mahmoud et al., 2023), and surface electromyography (Liang et al., 2024) to assess the impact of BFRT on ankle muscle strength in patients with CAI. Three of these studies (Werasirirat and Yimlamai, 2022; Wen et al., 2023; Liu and Wang, 2024) indicated that BFRT effectively enhanced inversion, eversion, plantarflexion, and dorsiflexion strength by the conclusion of the intervention period. Notably, Wen et al. (2023) found no significant differences in strength improvements between the BFRT group and the conventional training group, whereas Mahmoud et al. (2023) reported more substantial enhancements in the BFRT group. In contrast, Liu and Wang (2024) observed that BFRT outperformed conventional interventions only in improving dorsiflexion and plantarflexion strength. Another study (Werasirirat and Yimlamai, 2022) similarly reported significant improvements in eversion, plantarflexion, and dorsiflexion strength in the BFRT group, with superior gains in eversion and plantarflexion compared to conventional training. In addition, a study (Liang et al., 2024) found that BFRT also significantly elevated the maximal isometric strength of the tibialis anterior and lateral head of the gastrocnemius muscles in CAI patients.

#### 3.5.4 Effects of BFRT on balance

Of the nine included studies, eight investigated the effects of BFRT on balance in patients with CAI. These studies assessed dynamic balance using the Y Balance Test (YBT) (Burkhardt et al., 2021; Werasirirat and Yimlamai, 2022; Wen et al., 2023; Liang et al., 2024; Liu S. et al., 2024), side hop test (SHT) (Werasirirat and Yimlamai, 2022), Biodex Balance System (Mahmoud et al., 2023), and star excursion balance test (SEBT) (Clark et al., 2024), while static balance was evaluated using the single-leg stance with eyes closed test (Liu S. et al., 2024). Ankle stability was measured through the Cumberland Ankle Instability Tool (CAIT) (Liang et al., 2024; Liu S. et al., 2024; Liu and Wang, 2024). However, the results varied across studies.

Seven studies (Burkhardt et al., 2021; Werasirirat and Yimlamai, 2022; Mahmoud et al., 2023; Wen et al., 2023; Liang et al., 2024; Liu S. et al., 2024; Liu and Wang, 2024) reported that single-session or short-to mid-term BFRT significantly improved balance in CAI patients. Among these, four studies (Werasirirat and Yimlamai, 2022; Wen et al., 2023; Liang et al., 2024; Liu S. et al., 2024) found that the improvements were comparable to those observed in conventional training groups, while three studies (Burkhardt et al., 2021; Mahmoud et al., 2023; Liu and Wang, 2024) demonstrated that BFRT yielded superior effects compared to conventional training. Only one study (Clark et al., 2024) reported that patients with CAI showed lower composite Star Excursion Balance Test (SEBT) scores and greater perceived instability during dynamic balance exercises with BFRT compared to conventional training. Table 3 shows the intervention characteristics in detail.

**TABLE 3:** Intervention characteristics.

Study	Intervention (EG/CG)	BFRT pressure, width, placement	Intensity	Duration and frequency	Exercise mode	Outcome extracted	Limitations
Killinger et al. (2020)	BFRT + RR/RR	80%LOP; 11 cm; proximal thigh	30%MVIC	Single practice	Ankle eversion and dorsiflexion exercises(4 sets × 30/15/15/15)	MA (TA ↑,PL-); SmO_2_ ↑; RPE ↑	Limited generalizability Restricted clinical applicability; Unverified long-term utility
Burkhardt et al. (2021)	BFRT + RR/RR	80%AOP; 11 cm; proximal knee	20%–40%1RM	2 tests in 24–48 h	YBT(4 sets × 30/15/15/15)	MA (gastrocnemius ↑, TA-, PL-); YBT ↑	Limited generalizability Acute effects only Narrow metrics
Werasirirat and Yimlamai (2022)	BFRT + RR/RR	80%AOP; 10 cm; proximal thigh	≤20%1RM	6 weeks; 3times/wk	Heel raise exercise(4 sets × 30/15/15/15); leg squats(3 sets × 10 reps); YBT(1 set ×5 reps)	MS ↑ (inversion, eversion, plantarflexion, and dorsiflexion); CSA (PL ↑); SHT ↑; YBT ↑	Limited study design Small sample size Unexplained mechanisms
Wen et al. (2023)	BFRT + RR/RR	80%AOP; 14 cm; proximal knee	20%–40%1RM	6 weeks; 3times/wk	Ankle inversion, eversion, plantarflexion, and dorsiflexion exercises(4 sets × 30/15/15/15)	MS ↑ (inversion, eversion, plantarflexion, and dorsiflexion); CSA(TA ↑, PL ↑, TS ↑); YBT ↑	Uncontrolled activity confounders; Lack of subjective functional assessment Limited generalizability (to High Pain)
Mahmoud et al. (2023)	BFRT/BFRT + RR, RR	80%AOP; 10 cm; proximal thigh	20%–40%1RM	4 weeks; 3times/wk	EG: 5-min compression stimulation (1 session/day, 5 sets/session). CG: elastic band resistance training targeting ankle inversion, eversion, plantarflexion, and dorsiflexion (3 sets × 10 reps); Balance board training (10 reps × 15 s)	MS ↑ (inversion, eversion, plantarflexion, and dorsiflexion); YBT ↑	Limited generalizability (Female-only cohort)
Liu S. et al. (2024)	BFRT + RR/RR	7 levels of subjective pressure; 5 cm; proximal thigh	Low-Load	4 weeks; 3times/wk	Ankle inversion, eversion, plantarflexion, and dorsiflexion exercises(3 sets × 10 reps); Balance mat exercises (3 sets × 1 min); ROM (6 sets × 30 s)	CAIT ↑; MA (TA ↑, PL-); YBT ↑	Limited muscle focus Non-functional sEMG setting Small sample and Short duration
Clark et al. (2024)	BFRT + RR/RR	80%AOP; NR; proximal thigh	Low-Load	2 tests in 24–48 h	SEBT(4 sets × 30/15/15/15)	SEBT ↓; RPI ↑; RPE ↑	Missing baseline measures Limited generalizability
Liu and Wang (2024)	BFRT + RR/RR	20–50 mmhg; 10 cm; NR	Low-Load	4 weeks; 2times/wk	Heel raise exercise(4–6 sets ×12–15 reps); Ankle inversion, eversion,and dorsiflexion exercises(6 sets ×10–20 reps); Bosu ball(1–6 sets of reps)	MS ↑ (inversion, eversion, plantarflexion, and dorsiflexion); CAIT ↑	Small sample; Short duration Narrow population Subjective measures
Liang et al. (2024)	BFRT + RR/RR	60%AOP; 11 cm; proximal thigh	Low-Load	6 weeks; 3times/wk	Single-leg forward/backward jumps, lateral jumps, and two-legged vertical jumps (4 sets × 10 reps); single-leg quadrangle, Z-shaped, and vertical jumps (4 sets × 15 reps); IASTM(Before BFRT)	MA and MS (gastrocnemius ↑,TA ↑); YBT ↑; CAIT ↑	Absence of follow-up

EG, experimental group; CG, control group; RR, routine rehabilitation; 1RM, one-repetition maximum; MVIC, maximum voluntary isometric contraction; TA, tibialis anterior; PL, peroneus longus; TS, triceps surae; LOP, limb occlusion pressure; AOP, arterial occlusion pressure; MVIC, maximum voluntary isometric contraction; BFRT, blood flow restriction training; YBT, Y Balance Test; CAIT, the cumberland ankle instability tool; SEBT, star excursion balance test; IASTM, instrument-assisted soft tissue mobilization; SmO_2_, muscle oxygen saturation; MS, muscle strength; MA, muscle activation; CSA, muscle cross-sectional area; SHT, side hop test; RPI, rating of perceived instability; RPE, rating of perceived exertion; ↑ represents a significant increase; ↓represents a significant decrease; - represents no significant change; NR, not reported.

## 4 Discussion

The purpose of this systematic review was to explore the effects of BFRT on muscle function and balance in patients with CAI. Findings demonstrate that BFRT combined with low-intensity conventional rehabilitation training significantly enhances ankle muscle strength and promotes muscle hypertrophy in patients with chronic ankle instability (CAI). Despite variations in training protocols and BFRT parameters across studies, these benefits have been consistently observed in both acute (single-treatment) and short-term (4–6 weeks) interventions. Furthermore, most of the included studies support its significant effects on improving muscle activation and balance, but some of the research results are still inconsistent.

Among the key muscle groups involved in the synergistic contraction of the ankle joint, the PL and TA play critical roles in ankle inversion and dorsiflexion, respectively, and are primarily responsible for regulating lateral ankle stability (Tashiro et al., 2021; Hyodo et al., 2022). The TS, comprising the gastrocnemius and soleus muscles, works synergistically in toe flexion and plantarflexion, and is crucial for maintaining overall ankle stability (Kim et al., 2012). However, in patients with CAI, reduced muscle strength around the ankle impairs this synergistic contraction, leading to diminished joint stability (Liu et al., 2022). In our study, we found that BFRT combined with low-intensity rehabilitation training significantly enhanced the strength of inversion, eversion, plantarflexion, and dorsiflexion muscles in CAI patients. Its effect was comparable to that of conventional rehabilitation training, and in some specific muscle groups, the improvements were even more pronounced. This outcome aligns with findings from studies on the hip and knee (Van Cant et al., 2020; Miller et al., 2021; Constantinou et al., 2022). Moreover, while enhancing ankle muscle strength, low-intensity BFRT also significantly promoted hypertrophy in the PL, TA, and TS muscle groups, supporting the well-established positive correlation between muscle strength and muscle thickness (Miyachi et al., 2022). Therefore, the enhancement of muscle strength by BFRT in CAI patients may be achieved by increasing the cross-sectional area of muscle fibers. The potential mechanism may be due to the fact that BFRT triggers the accumulation of metabolites in a hypoxic environment, leading to cellular swelling, which promotes protein synthesis and inhibits protein hydrolysis, ultimately inducing muscle hypertrophy (Martin et al., 2022).

The degree of muscle activation reflects the ability of muscles to recruit motor units and reflects the control effect of the central nervous system on the stability of lower limb joints (Burkhardt et al., 2021). Three studies (Killinger et al., 2020; Liang et al., 2024; Liu S. et al., 2024) have shown that low-intensity BFRT significantly increased the level of TA activation in patients with CAI, and two other studies have found that its activation of the gastrocnemius muscle (Liang et al., 2024) and the soleus muscle (Burkhardt et al., 2021) also showed a similar improvement. This may be due to the accumulation of metabolites in the muscle under the ischemic and hypoxic environment after blood flow restriction, which inhibits the recruitment of low-threshold type I motor units and mobilizes high-threshold type II motor units (Burkhardt et al., 2021). This mechanism could also contribute to the observed increase in ankle muscle strength among CAI patients. However, the study by Burkhardt et al. (2021) did not find an intervention effect of BFRT on TA activation, which may be due to the differences in metabolic demands on the muscles, depending on the type of exercise (Burkhardt et al., 2021). Additionally, none of the four studies (Killinger et al., 2020; Burkhardt et al., 2021; Liang et al., 2024; Liu S. et al., 2024) investigating the effects of BFRT on muscle activation in CAI patients found a significant improvement in PL activation. This could be because the PL, as a small muscle with a unique anatomical position, may require specifically targeted training for effective activation (Liang et al., 2024). The above study demonstrated that BFRT significantly improved muscle function in CAI patients and induced greater neuromuscular adaptations with lower exercise loads (Yang et al., 2022). However, it is important to note that combining BFRT with different types of exercises may influence the activation of peri-ankle muscles.

In addition to significantly improving muscle function in CAI patients, seven studies (Killinger et al., 2020; Burkhardt et al., 2021; Werasirirat and Yimlamai, 2022; Mahmoud et al., 2023; Wen et al., 2023; Liang et al., 2024; Liu S. et al., 2024; Liu and Wang, 2024) also found that low-intensity BFRT had a notable effect on improving their balance. This improvement is likely closely related to the enhancement of muscle function. Firstly, it has been well-documented that muscle strength and muscle mass are positively correlated with balance ability (Hall et al., 2015; Kim and Jeon, 2016). As a result, the improvement in balance observed in CAI patients may be directly linked to the increased strength in peri-ankle muscle groups such as the PL, TA, and TS. Secondly, BFRT may enhance neuromuscular control of the ankle joint by increasing the activation levels of these muscle groups, reducing the myoelectric delay of the muscle spindle stretch reflex, and enabling the body to more rapidly adjust limb control during shifts in the center of gravity (Hall et al., 2015). However, one study did not find a significant improvement in balance with BFRT in CAI patients, which might be attributed to reduced knee mobility resulting from quadriceps overuse during training (Clark et al., 2024). Furthermore, Killinger et al. (2020) similarly observed that CAI patients in the BFRT group experienced greater fatigue during dynamic balance exercises. This fatigue may arise from localized hypoxia and metabolite accumulation during ischemic exercise, which disrupts neuronal ion channel function, impairs nerve conduction, lowers motor neuron firing rates, and ultimately diminishes muscle activation (Wang et al., 2025). Additionally, training parameters—including load intensity, rest intervals, and cuff pressure—modulate the development of fatigue (Wang et al., 2025). This phenomenon has been corroborated by other studies (Rivera et al., 2023), suggesting that the fatigue induced by BFRT may negatively affect postural stability in CAI patients. Such effects are likely mediated through fatigue-related impairments in proprioception, sensorimotor integration, and neuromuscular control (Choi and Lee, 2020).

Additionally, only one of the nine studies included in this review demonstrated that BFRT significantly reduced the recurrence rate of ankle injuries after 1 year of muscle strength and balance improvements in CAI patients. However, the follow-up sample size in this study was small (Liu S. et al., 2024). The remaining eight studies did not examine the long-term effects of BFRT on muscle function and balance in CAI patients. Notably, all included studies exhibited considerable heterogeneity in blood flow restriction parameters—such as pressure setting, duration, and cuff placement—as well as in training protocols. Methodologically, this variability precludes definitive attribution of the observed effects on muscle function and balance to any single intervention factor. Nevertheless, the available evidence provides robust synthesized support for the overall findings of this review.

This review has several limitations. First, the variability in exercise protocols (e.g., type, frequency, intensity) and BFRT parameters (e.g., pressure settings, duration, cuff position) reduces the comparability across studies, limiting the ability to draw firm conclusions. Second, all studies included young adults (mean age ∼20 years), which limits the generalizability of the findings.

## 5 Conclusion

Combining BFRT with low-intensity conventional rehabilitation, during either a single acute BFRT session or a 4- to 6-week intervention period, significantly enhances ankle muscle strength and promotes muscle hypertrophy in CAI patients. Furthermore, most studies highlight BFRT’s positive effects on muscle activation and balance function. However, some findings remain inconsistent and require further investigation.

To strengthen the evidence base, future research should prioritize the following: 1) establishing standardized BFRT protocols for CAI that define key parameters such as cuff placement, restriction pressure, and load intensity; 2) conducting longitudinal studies extending beyond 6 months to assess long-term outcomes, including rates of ankle sprain recurrence; 3) and expanding investigations to include diverse populations, such as athletes and older adults, through multicenter randomized controlled trials. These efforts will help elucidate the applicability and optimization of BFRT across clinical and athletic contexts.

## Data Availability

The original contributions presented in the study are included in the article/supplementary material, further inquiries can be directed to the corresponding author.

## References

[B1] Anguish B. Sandrey M. A. (2018). Two 4-week balance-training programs for chronic ankle instability. J. Athl. Train. 53 (7), 662–671. 10.4085/1062-6050-555-16 30192681 PMC6138271

[B2] Burkhardt M. Burkholder E. Goetschius J. (2021). Effects of blood flow restriction on muscle activation during dynamic balance exercises in individuals with chronic ankle instability. J. Sport Rehabil. 30 (6), 870–875. 10.1123/jsr.2020-0334 33547257

[B3] Cao S. Liu J. Wang Z. Geok S. K. (2024). The effects of functional training on physical fitness and skill-related performance among basketball players: a systematic review. Front. Physiol. 15, 1391394. 10.3389/fphys.2024.1391394 38784117 PMC11112112

[B4] Cashin A. G. McAuley J. H. (2020). Clinimetrics: physiotherapy evidence database (pedro) scale. J. Physiother. 66 (1), 59. 10.1016/j.jphys.2019.08.005 31521549

[B5] Choi H. S. Lee J. H. (2020). Immediate effect of balance taping using kinesiology tape on dynamic and static balance after ankle muscle fatigue. Healthcare 8 (2), 162. 10.3390/healthcare8020162 32526892 PMC7348943

[B6] Clark K. Trickett J. Donovan L. Dawson J. Goetschius J. (2024). Effects of blood flow restriction on balance performance during dynamic balance exercises in individuals with chronic ankle instability. J. Sport Rehabil. 33 (3), 181–188. 10.1123/jsr.2023-0182 38350443

[B7] Constantinou A. Mamais I. Papathanasiou G. Lamnisos D. Stasinopoulos D. (2022). Comparing hip and knee focused exercises versus hip and knee focused exercises with the use of blood flow restriction training in adults with patellofemoral pain. Eur. J. Phys. Rehabil. Med. 58 (2), 225–235. 10.23736/S1973-9087.22.06691-6 34985237 PMC9980495

[B8] Delahunt E. Remus A. (2019). Risk factors for lateral ankle sprains and chronic ankle instability. J. Athl. Train. 54 (6), 611–616. 10.4085/1062-6050-44-18 31161942 PMC6602396

[B9] Doherty C. Delahunt E. Caulfield B. Hertel J. Ryan J. Bleakley C. (2014). The incidence and prevalence of ankle sprain injury: a systematic review and meta-analysis of prospective epidemiological studies. Sports Med. 44 (1), 123–140. 10.1007/s40279-013-0102-5 24105612

[B10] Gribble P. A. Bleakley C. M. Caulfield B. M. Docherty C. L. Fourchet F. Fong D. T. (2016). Evidence review for the 2016 international ankle consortium consensus statement on the prevalence, impact and long-term consequences of lateral ankle sprains. Br. J. Sports Med. 50 (24), 1496–1505. 10.1136/bjsports-2016-096189 27259753

[B11] Hall E. A. Docherty C. L. Simon J. Kingma J. J. Klossner J. C. (2015). Strength-training protocols to improve deficits in participants with chronic ankle instability: a randomized controlled trial. J. Athl. Train. 50 (1), 36–44. 10.4085/1062-6050-49.3.71 25365134 PMC4299733

[B12] Hall E. A. Chomistek A. K. Kingma J. J. Docherty C. L. (2018). Balance- and strength-training protocols to improve chronic ankle instability deficits, part ii: assessing patient-reported outcome measures. J. Athl. Train. 53 (6), 578–583. 10.4085/1062-6050-387-16 29995462 PMC6089028

[B13] Hertel J. Corbett R. O. (2019). An updated model of chronic ankle instability. J. Athl. Train. 54 (6), 572–588. 10.4085/1062-6050-344-18 31162943 PMC6602403

[B14] Hwang P. S. Willoughby D. S. (2018). Mechanisms behind blood flow-restricted training and its effect toward muscle growth. J. Strength and Cond. Res. 33 (Suppl. 1), S167-S179–S179. 10.1519/jsc.0000000000002384 30011262

[B15] Hyodo Y. Jiroumaru T. Kida N. Wachi M. Nomura S. Kuroda M. (2022). Elucidation of abductor digiti minimi activity in chronic ankle instability. J. Phys. Ther. Sci. 34 (3), 242–246. 10.1589/jpts.34.242 35291477 PMC8918096

[B16] Killinger B. Lauver J. D. Donovan L. Goetschius J. (2020). The effects of blood flow restriction on muscle activation and hypoxia in individuals with chronic ankle instability. J. Sport Rehabil. 29 (5), 633–639. 10.1123/jsr.2018-0416 31094639

[B17] Kim K. Jeon K. (2016). Development of an efficient rehabilitation exercise program for functional recovery in chronic ankle instability. J. Phys. Ther. Sci. 28 (5), 1443–1447. 10.1589/jpts.28.1443 27313347 PMC4905886

[B18] Kim K. M. Ingersoll C. D. Hertel J. (2012). Altered postural modulation of hoffmann reflex in the soleus and fibularis longus associated with chronic ankle instability. J. Electromyogr. Kinesiol. 22 (6), 997–1002. 10.1016/j.jelekin.2012.06.002 22795679

[B19] Kobayashi T. Gamada K. (2014). Lateral ankle sprain and chronic ankle instability: a critical review. Foot Ankle Spec. 7 (4), 298–326. 10.1177/1938640014539813 24962695

[B20] Korkmaz E. Donmez G. Uzuner K. Babayeva N. Torgutalp S. S. Ozcakar L. (2022). Effects of blood flow restriction training on muscle strength and architecture. J. Strength Cond. Res. 36 (5), 1396–1403. 10.1519/JSC.0000000000003612 32287091

[B21] Liang X. Han Y. Wang S. Pan W. Jiang Y. Wei X. (2024). Effect of blood flow restriction combined with low-intensity plyometric jump training on functional ankle instability. Chin. J. Rehabil. Theory Pract. 30 (03), 352–361. 10.3969/j.issn.1006-9771.2024.03.014

[B22] Lin C. I. Houtenbos S. Lu Y. H. Mayer F. Wippert P. M. (2021). The epidemiology of chronic ankle instability with perceived ankle instability-a systematic review. J. Foot Ankle Res. 14 (1), 41. 10.1186/s13047-021-00480-w 34049565 PMC8161930

[B23] Liu Y. Wang Y. (2024). Study on the effect of blood flow restriction training combined with iastam on ankle strength and function intervention in athletes with chronic ankle instability in sport dance events. BMC Sports Sci. Med. Rehabil. 16 (1), 81. 10.1186/s13102-024-00873-x 38605396 PMC11007892

[B24] Liu K. Delaney A. N. Kaminski T. W. (2022). A review of the role of lower-leg strength measurements in ankle sprain and chronic ankle instability populations. Sports Biomech. 21 (4), 562–575. 10.1080/14763141.2021.1912165 33938376

[B25] Liu H. Jiang L. Wang J. (2024). The effects of blood flow restriction training on post activation potentiation and upper limb muscle activation: a meta-analysis. Front. Physiol. 15, 1395283. 10.3389/fphys.2024.1395283 39055689 PMC11269198

[B26] Liu N. Yang C. Song Q. Yang F. Chen Y. (2024). Patients with chronic ankle instability exhibit increased sensorimotor cortex activation and correlation with poorer lateral balance control ability during single-leg stance: a fnirs study. Front. Hum. Neurosci. 18, 1366443. 10.3389/fnhum.2024.1366443 38736530 PMC11082417

[B27] Liu S. Tang J. Hu G. Xiong Y. Ji W. Xu D. (2024). Blood flow restriction training improves the efficacy of routine intervention in patients with chronic ankle instability. Sports Med. Health Sci. 6 (2), 159–166. 10.1016/j.smhs.2023.11.001 38708328 PMC11067764

[B28] Mahmoud W. S. Radwan N. L. Ibrahim M. M. Hasan S. Alamri A. M. Ibrahim A. R. (2023). Effect of blood flow restriction as a stand-alone treatment on muscle strength, dynamic balance, and physical function in female patients with chronic ankle instability. Med. Baltim. 102 (44), e35765. 10.1097/MD.0000000000035765 37933020 PMC10627705

[B29] Martin P. M. Bart R. M. Ashley R. L. Velasco T. Wise S. R. (2022). An overview of blood flow restriction physiology and clinical considerations. Curr. Sports Med. Rep. 21 (4), 123–128. 10.1249/JSR.0000000000000948 35394953

[B30] Miller B. C. Tirko A. W. Shipe J. M. Sumeriski O. R. Moran K. (2021). The systemic effects of blood flow restriction training: a systematic review. Int. J. Sports Phys. Ther. 16 (4), 978–990. 10.26603/001c.25791 34386277 PMC8329318

[B31] Miyachi R. Koike N. Kodama S. Miyazaki J. (2022). Relationship between trunk muscle strength and trunk muscle mass and thickness using bioelectrical impedance analysis and ultrasound imaging. Biomed. Mater. Eng. 33 (1), 31–40. 10.3233/BME-211218 34250924

[B32] Moseley A. M. Rahman P. Wells G. A. Zadro J. R. Sherrington C. Toupin-April K. (2019). Agreement between the cochrane risk of bias tool and physiotherapy evidence database (pedro) scale: a meta-epidemiological study of randomized controlled trials of physical therapy interventions. PLoS One 14 (9), e0222770. 10.1371/journal.pone.0222770 31536575 PMC6752782

[B33] Narici M. V. Maffulli N. (2010). Sarcopenia: characteristics, mechanisms and functional significance. Br. Med. Bull. 95 (1), 139–159. 10.1093/bmb/ldq008 20200012

[B34] Rivera P. M. Proppe C. E. Gonzalez-Rojas D. Wizenberg A. Hill E. C. (2023). Effects of load matched isokinetic versus isotonic blood flow restricted exercise on neuromuscular and muscle function. Eur. J. Sport Sci. 23 (8), 1629–1637. 10.1080/17461391.2023.2184724 36825621

[B35] Tashiro T. Maeda N. Sasadai J. Kotoshiba S. Sakai S. Suzuki Y. (2021). Tensiomyographic neuromuscular response of the peroneus longus and tibialis anterior with chronic ankle instability. Healthcare 9 (6), 707. 10.3390/healthcare9060707 34200684 PMC8230383

[B36] Vallandingham R. A. Gaven S. L. Powden C. J. (2019). Changes in dorsiflexion and dynamic postural control after mobilizations in individuals with chronic ankle instability: a systematic review and meta-analysis. J. Athl. Train. 54 (4), 403–417. 10.4085/1062-6050-380-17 30870009 PMC6522095

[B37] Van Cant J. Dawe-Coz A. Aoun E. Esculier J. F. (2020). Quadriceps strengthening with blood flow restriction for the rehabilitation of patients with knee conditions: a systematic review with meta-analysis. J. Back Musculoskelet. Rehabil. 33 (4), 529–544. 10.3233/BMR-191684 32310159

[B38] Wang J. Xu J. Liu H. Jiang L. (2025). The immediate effects of blood flow restriction training on upper limb muscle strength and fatigue level: a meta-analysis. Front. Physiol. 16 (1), 1521145. 10.3389/fphys.2025.1521145 40666112 PMC12259574

[B39] Wen Z. Zhu J. Wu X. Zheng B. Zhao L. Luo X. (2023). Effect of low-load blood flow restriction training on patients with functional ankle instability: a randomized controlled trial. J. Sport Rehabil. 32 (8), 863–872. 10.1123/jsr.2022-0462 37558223

[B40] Werasirirat P. Yimlamai T. (2022). Effect of supervised rehabilitation combined with blood flow restriction training in athletes with chronic ankle instability: a randomized placebo-controlled trial. J. Exerc. Rehabil. 18 (2), 123–132. 10.12965/jer.2244018.009 35582686 PMC9081407

[B41] Yang S. Zhang P. Sevilla-Sanchez M. Zhou D. Cao J. He J. (2022). Low-load blood flow restriction squat as conditioning activity within a contrast training sequence in high-level preadolescent trampoline gymnasts. Front. Physiol. 13, 852693. 10.3389/fphys.2022.852693 35770193 PMC9234321

[B42] Yin Y. Yu Z. Wang J. Sun J. (2022). Effectiveness of the rehabilitation training combined with maitland mobilization for the treatment of chronic ankle instability: a randomized controlled trial. Int. J. Environ. Res. Public Health 19 (22), 15328. 10.3390/ijerph192215328 36430049 PMC9690276

